# Exploring Heterozygosity-Survival Correlations in a Wild Songbird Population: Contrasting Effects between Juvenile and Adult Stages

**DOI:** 10.1371/journal.pone.0105020

**Published:** 2014-08-14

**Authors:** David Canal, David Serrano, Jaime Potti

**Affiliations:** 1 Doñana Biological Station – CSIC, Department of Evolutionary Ecology, Sevilla, Spain; 2 Doñana Biological Station – CSIC, Department of Conservation Biology, Sevilla, Spain; University of Massachusetts, United States of America

## Abstract

The relationship between genetic diversity and fitness, a major issue in evolutionary and conservation biology, is expected to be stronger in traits affected by many loci and those directly influencing fitness. Here we explore the influence of heterozygosity measured at 15 neutral markers on individual survival, one of the most important parameters determining individual fitness. We followed individual survival up to recruitment and during subsequent adult life of 863 fledgling pied flycatchers born in two consecutive breeding seasons. Mark-recapture analyses showed that individual heterozygosity did not influence juvenile or adult survival. In contrast, the genetic relatedness of parents was negatively associated with the offspring’s survival during the adult life, but this effect was not apparent in the juvenile (from fledgling to recruitment) stage. Stochastic factors experienced during the first year of life in this long-distance migratory species may have swamped a relationship between heterozygosity and survival up to recruitment.

## Introduction

Mating between related individuals may entail fitness costs to their descendents (e.g. [1]–[3]). The relationship between genetic diversity and fitness has therefore received much attention due to its potential importance in animal production, conservation and evolutionary biology (review in [4]–[9]). The study of this relationship in natural populations has traditionally been complex due to the difficulty of generating pedigrees to estimate individual coancestry [4]. With the expansion of molecular techniques in the last decades, however, an alternative approach based on the relationship between genetic diversity measured at a set of loci and traits related to fitness (heterozygosity-fitness correlation, HFC) has become widespread in the literature (review in [5], [6], [10]).

Positive HFCs, the association most commonly found in the literature [6], may arise mainly through three mechanisms [9], [11]. Under the “direct hypothesis” heterozygous individuals have higher fitness due to overdominance of the typed loci. This mechanism may be important when heterozygosity is measured with allozymes or at the major histocompatibility complex (MHC) loci, but does not explain HFC in studies using microsatellites which, aside from exceptions (e.g. [12]), are considered neutral loci [13]. When employing microsatellites, two alternative hypotheses have been proposed to explain HFCs. First, the “local effect hypothesis” predicts that HFCs arise indirectly because the typed loci are linked to functional loci influencing fitness. Genetic drift, migration and selection generate linkage favoring the detection of local effects [3], [10]. Although linkage is expected to be low and rapidly eroded by recombination in natural populations [3], [10], previous work has shown that relatively high levels of linkage may exist [14], [15] and be maintained after more than 800 generations following a bottleneck in natural populations [14]. Second, under the “general effect hypothesis”, heterozygosity measured at multiple loci (MLH) would reflect heterozygosity across the genome. In such cases, homozygous individuals suffer fitness costs due to their higher likelihood of expression of deleterious recessive alleles (inbreeding depression; [1], [2]). This mechanism is expected to arise under random mating, in populations with genetic drift, population admixture or suffering from recent bottlenecks, or in large populations where consanguineous matings occur. Populations under these conditions exhibit a large variance in inbreeding values, which determine the strength of the relationship between MLH and fitness [16], [17].

Evidence of HFCs is widespread but also inconsistent in the literature (e.g. [18]–[23]). In general, HFCs are weak signals explaining no more than 3.6% of the variance in fitness [6]. Nevertheless, the magnitude of HFC may depend on the characteristics of the population (see above), the traits under scrutiny, and the environmental conditions that individuals experience [10], [17], [24]. HFCs have been commonly explored in behavioral (e.g. post-fledgling dispersal: [25]) or morphological traits (e.g. body size: [26]; attractiveness: [27]), which are often under stabilizing selection [28]. However, evolutionary theory predicts [28], and empirical work confirms [5], [6], greater HFCs in fitness-related traits under directional selection, as is the case of life history traits (e.g. fecundity, lifetime reproductive success, survival; [10]). This is because such traits are affected by multiple loci susceptible of deleterious recessive mutations [10]. Survival is one of the most important factors determining individual fitness and evidence of heterozygosity-survival correlations (HSC, hereafter) is common [19], [29], [30]. In birds, there is increasing information on HSC during early life (embryonic and nestling stages, or up to recruitment: e.g. [6], [18], [31]). Nevertheless, in the wild, HSC has only been explored beyond those stages, to our knowledge, in a Seychelles warbler (*Acrocephalus sechellensis*; [32], [33]) and a blue tit (*Cyanistes caeruleus*; [12]) population.

HFCs are expected to decrease with age since differences in survival are greatest in early life [11]. Comprehensive studies across the lifespan of individuals, however, are crucial to the understanding of the mechanisms underlying HFCs since, for example, inbreeding effects may be underestimated [34], [35] or even undetectable [36] when analyzed at a single stage. In addition, MLH effects may be negative early in life but positive during adult life [12]. In combination with age, the role of genetic diversity on fitness may also be sensitive to the environmental conditions that individuals experience [4], [24]. Accordingly, recent studies highlight that, as a consequence of context-dependence, the magnitude of HFC may be inconsistent across years [37] or undetectable under favorable environmental conditions [33], [38], i.e. if variance in the measured trait is affected by the environment, HFCs will be more easily detected in periods when environmental conditions cause large variation in the trait.

Here, we investigated the relationship between heterozygosity and individual survival across different life stages in a population of pied flycatchers (*Ficedula hypoleuca*), a long-distance passerine migrant. We followed individuals from two consecutive cohorts throughout their entire lifespan and explore individual survival using capture-recapture methods. This modeling framework is a more robust approach than generalized linear models when exploring effects on survival in open populations because it accounts for resighting probabilities [39], [40]. Specifically, we explored whether: i) variation in survival was influenced by individual heterozygosity (MLH and/or single loci heterozygosity) or ii) by the genetic similarity or heterozygosity of an individual’s parents, and iii) MLH and/or the magnitude of HFC changed across lifetime [11]. Thus, this study provides a suitable opportunity to investigate the influence of individual heterozygosity on survival in a wild population.

## Materials and Methods

### Ethics Statement

We hereby confirm that all data came from authorized monitoring of a natural population of pied flycatchers *Ficedula hypoleuca* studied in La Hiruela (province of Madrid, Spain, coordinates 41° 04′ N, 3° 27′ E) since 1984. Such long-term study requires birds being subjected to minimal disturbance and trying to keep animal suffering to a minimum. Manipulation was restricted to blood extraction from the brachial vein and all birds recovered well and were not released until we assessed their welfare. All fieldwork was conducted with the required permits for capture, ringing and blood sampling of birds by Consejería de Medio Ambiente, Comunidad de Madrid and Delegación de Medio Ambiente, Junta de Castilla-La Mancha.

### Field work and general procedures

The study was carried out with individuals born in the breeding season of 2005 and 2006 as part of a long-term study of pied flycatchers in central Spain (e.g., [41], [42]). The study area consists of two plots separated by 1.3 km, including 236 nest-boxes. Field protocols have been described in detail elsewhere [41]. Briefly, all nests were regularly checked every three days before the onset of egg laying and on a daily basis around hatching to ascertain laying date, clutch size, hatching date and number of fledglings. Parent birds were captured with a nest-box trap while feeding eight day-old nestlings. They were weighed, measured and individually marked with a numbered metal band and a unique combination of colored bands. Fledglings were banded, measured and weighed at 13 days of age. Blood samples were taken from all fledglings by puncturing the brachial vein and stored in absolute ethanol. Sex determination was carried out by PCR amplification of the CHD gene using the primers 2917 (forward) and 3088 (reverse; [43]). Molecular sexing was always fully consistent with the sex of recruited individuals.

Apparent survival of individual offspring was assessed through an extensive effort of marking, recapturing and resighting of color-banded birds in all subsequent breeding seasons until 2013. This population has high natal philopatry with a mean of 13% of recruitment of locally born birds, which is the highest recruitment rate found for the species [42], [44], [45].

### Molecular methods

Our data set comprised 863 individuals born in 2005 (n = 235) and 2006 (n = 628), of which 101 recruited as breeders. Fledglings were genotyped at 15 polymorphic microsatellite loci: *f3-60, f1-25* [46], *fhy 216, fhy 237, fhy 301, fhy 304, fhy 310, fhy 329, fhy 339, fhy 356, fhy 361, fhy 401, fhy 444, fhy 466* and *fhy 236* [47]. Most individuals (n = 835; 96%) were genotyped at all loci, whereas genotyping failed for 23 individuals (2.6%) at one locus and for 5 individuals (0.6%) at two loci, respectively. Five individuals (0.6%) were discarded for further analyses since they were not genotyped for 4 or more loci.

Tests for Hardy-Weinberg equilibrium and linkage disequilibrium were done using the genotypes from the adult population of each study year with program Genepop 4.0 [48]. We performed a search in the zebra finch (*Taeniopygia guttata*) genome [49] to find the chromosome location of the loci used. A BLAT and BLAST search were run in UCSC (http://genome.ucsc.edu/) and ENSEMBL (http://www.ensembl.org/Taeniopygia_guttata/blastview) browsers, respectively, to confirm the locations of the sequences. The best matched sequence was selected on the basis of both the lowest *E-value* and highest score. The “contig view” option in ENSEMBL was used to locate the nearest gene to the best matching sequence.

The set of markers used allowed us to identify cases of extra pair paternity and correct for it when exploring a paternal effect on fledgling survival (see below). Parentage analyses were carried out on CERVUS 3.0 [50] using a maximum likelihood method (see [41], [51] for details). We considered a given male as the sire when he had a LOD (natural logarithm of the likelihood) score with a fledgling higher than the critical value requested for assignments at 95% confidence level (critical value is computed by CERVUS through parentage analyses simulations).

### Estimation of heterozygosity and identity disequilibrium

Multilocus individual heterozygosity and allele frequencies were calculated with the Excel macro Cernicalin [52]. Cernicalin calculates three metrics: observed homozygosity per individual (HO), Internal relatedness (IR) and homozygosity by loci (HL). The three metrics were highly correlated (n = 863, all r>0.97, p<0.001) and results did not vary among metrics (not shown). Analyses with HL are reported here because HL correlates better with genome-wide homozygosity and inbreeding in open populations than do other metrics [52]. HL varies between 0, when all loci in the individual are heterozygous, and 1, when all loci are homozygous. Homozygosity at a single locus (SHL) was coded as “0” for heterozygous status and “1” for homozygous. Pairwise relatedness coefficients of adults pairs (a proxy for inbreeding) were calculated through maximum likelihood estimation with software ML-RELATE [53].

Identity disequilibrium, the positive correlation between heterozygosity across loci, which is expected when HL is related to wide-genome heterozygosity, was calculated as g_2_ in the program RMES [54]. Genotypes were resampled 1000 times to test if g_2_ differed significantly from zero.

### Capture-Recapture models

We used standard capture-mark-recapture (CMR) models implemented in software MARK 6.0 [55] to estimate apparent survival (φ) and recapture (*p*) probabilities [39] with maximum likelihood techniques (e.g. [56]). In particular, an extension of the Cormack-Jolly-Seber model including age and group effects was used [39]. By estimating (and correcting for) recapture probabilities, these models provide less-biased estimates of true survival probabilities than return rates, especially when site propensity and/or true detection are low (e.g. [57], [58]). The recapture probability is the probability that a marked individual that is alive and present in the study area at sampling occasion *t* is captured (or observed) at sampling occasion *t*. The survival probability is the probability that a marked individual alive at sampling occasion *t* survived and has not permanently emigrated between sampling occasions *t* and *t+*1. These models make a number of assumptions to fit the available capture-recapture data. We tested the goodness of fit (GOF) of our data using U-CARE 2.3.2 [59].

In a first step, we tried to obtain a good fitting model in which potential genetic effects were not considered. Based on our previous knowledge of the species, four factors that could affect survival and recapture probabilities were included: time, sex, age (fledgling, 1-yr, 2-yr and older) and cohort (2005 versus 2006). We began with a global model in which all parameters were different (denoted φ_s*t*c_ *p*_s*t*c_, were s = sex, t = time and c = cohort). Given that age and time are identical within a given cohort, the three factors cannot be tested simultaneously, so we built plausible pairwise combinations of the three variables. We tried to constrain these initial models by building a set of candidate models with fewer parameters that had a reasonable biological justification (i.e. reducing the number of age-classes and assuming no differences among years and/or between sexes and cohorts). Once the most parsimonious models were identified, we added genetic data (genetic relatedness of parents and multilocus heterozygosity of fledglings and parents) as individual covariates to test their importance on apparent survival. Model selection was based on the Akaike’s information criterion adjusted for small sample sizes (AICc) and Akaike weights (*wi*, [60]). In addition, we used the analysis of deviance test (ANODEV, [61]) to compare the amount of deviance explained by a covariate.

To test for single locus effects on survival, we additionally built in a generalized linear modeling framework (GLMM with a binomial error distribution) i) a model including all single locus heterozygosities (SHL), and ii) a model including the individual HL, and compared them with a F-ratio test as suggested by Szulkin et al. [62]. In the model containing heterozygosities at all single loci, missing data at one locus were replaced by the sample average at that locus [62]. The procedure was performed for both juvenile and adult survival regardless of whether or not general effects were found. In fact, significant SLH effects may exist even in absence of HL correlation as loci may have similar effects in opposite directions [22]. Sample sizes varied slightly across statistical analyses because all information was not always available for all individuals.

## Results

### General genetic parameters

Individual HL ranged from 0 to 0.56, with a mean (±SD) of 0.208 (±0.102). HL did not differ between years (2005, n = 234: 0.198±0.102; 2006, n = 620: 0.212±0.102, GLMM: F_1,666_ = 1.10, p = 0.29), sex (males, n = 477: 0.205±0.103; females, n = 377: 0.212±0.100, GLMM: F_1,156_ = 0.68, p = 0.40) or among age groups (fledglings: 0.21±0.1; yearlings: 0.20±0.09; older individuals: 0.20±0.09; F_1,156_ = 0.22, p = 0.64; interaction age*sex: F_1,154_ = 0.05, p = 0.78). Nestlings’ HL and their parent relatedness were weakly but significantly correlated (r = 0.32; p<0.001).

The search within the zebra finch putative genome showed that the markers we used were widespread throughout the passerine genome (Table 1). All loci conformed to Hardy-Weinberg equilibrium and no pair of loci showed significant linkage disequilibrium after Bonferroni correction. Identity disequilibrium was low and did not differ significantly from zero (g_2_ = 0.0006, sd = 0.0008, p = 0.17).

**Table 1:** Characteristics of the microsatellite loci used in the study.

Locus	A	Ho	He	Chromosome: Start (Bp)	ID nearest gene*	Distance (Bp)
f1-25	7	0.738	0.7554	20/10.407.082	07700	exon+
f3-60	35	0.9543	0.961	9/14.479.023	09067	27.807
Fhy 216	8	0.518	0.521	1a/63.001.644	11914	11.2742
Fhy 236	25	0.896	0.87	20/13.791.266	08550	11.5271
Fhy 237	6	0.399	0.392	3/7.930.679	02614	15.700
Fhy 301	14	0.856	0.884	2/92.250.591	07400	150.859
Fhy 304	10	0.79	0.803	4_random/2.365.290	15203	763.363
Fhy 310	13	0.872	0.864	2/92.250.591	15081	2.257
Fhy 329	8	0.682	0.672	3/49.130.923	10789	68.805
Fhy 339	12	0.831	0.83	1/95.843.488	13407	3.561
Fhy 356	12	0.833	0.856	1a/6.627.591	02324	29.231
Fhy 361	7	0.549	0.518	2/29.361.136	01714	195.077
Fhy 401	13	0.795	0.788	Un/52.369.084	06481	696.145
Fhy 444	14	0.8757	0.8816	1/12.170.793	07097	293.505
Fhy 466	12	0.8362	0.8438	7/21.099.737	10012	5.274

Number of alleles (A), observed (Ho) and expected (He) heterozygosities, chromosome location, and distance to the nearest gene (in base pairs; Bp) according to their position in the zebra finch genome are shown.

*Last digits of the gene’s ID in ENSEMBL. Prefix: ENSTGUG000000.

+ located in a exon of a solute carrier organic anion transporter gene.

### Apparent survival

The U-CARE goodness of fit global test was highly significant (χ^2^ = 36.93, d.f. = 12, p = 0.0002). The specific test for transience was also significant (Z = 2.99, p = 0.001), while there was no statistical evidence for trap-dependence (Z = −1.23, p = 0.22). After suppressing the first occasion from the capture-recapture history, the GOF tests were no longer significant (Global test: χ^2^ = 6.21, d.f. = 8, p = 0.62; directional test for transience: Z = 1.16, p = 0.12), indicating that a true age effect was responsible for the lack of fit (i.e. the transient effect was associated with differential capture-recapture probability between juveniles and adults). We accounted for this in our models by estimating age-specific survival and recapture parameters.

The initial modelling process without genetic covariates revealed that two models received most support. The first model (φ_a2_, *p*_a3*s*c_, AICc = 1092.699, *K* = 11, *w_i_* = 0.398, Deviance = 1070.40) showed that survival was different for juveniles and adults, while recapture varied with time, and between sexes and cohorts. The second top-ranked model (φ_a2*s_, *p*_a3*s*c_, AICc = 1094.559, ΔAICc = 1.859, *K* = 13, *w_i_* = 0.157, Deviance = 1068.15) had the same structure but included a sex effect for apparent survival. The remaining models were separated by >4 AICc points and thus considered poorly supported (Results not shown).

**Figure 1:**
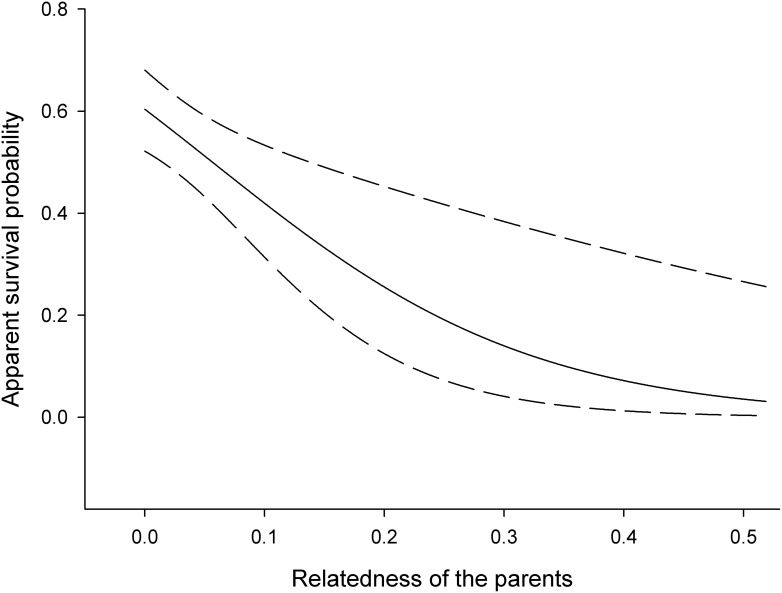
Apparent survival probability of adults (from their first-year onwards) in relation to the pairwise genetic relatedness of their parents. Low values indicate lower relatedness (e.g. relatedness between parent-offspring or full siblings is 0.5). Dashed lines denote 95% confidence limits around the predicted linear trend.

Table 2 shows the overall best models once the effect of genetic covariates was incorporated. The top-ranked model (model 1) indicated that relatedness of the parents affected offspring survival during adult life but not between fledgling and recruitment; parents with a higher relatedness coefficient raised fledglings that had lower survival probabilities throughout adulthood (Fig. 1). The covariate in this model has a highly significant effect (ANODEV test: *F*_1,17_ = 62.86, p<0.0001) and explained 78.7% of the survival variability. The second top-ranked model included also an effect on juvenile survival, but the examination of 95% confidence intervals for the estimate of the effect indicated that this parameter is confounding [60], [63]. Indeed, the model including survival from fledgling to recruitment only (model 13) received little support. In any case, models incorporating the effect of relatedness of the parents on survival had a cumulative weight of 0.977, being 43 times better supported than models without this effect. Models including the father’s (models 7, 9 and 11) or the mother’s (models 10 and 15) multilocus heterozygosity were hardly supported, as was a model with multilocus fledgling heterozygosity (model 14) or models without any covariate (models 8 and 12).

**Table 2:** Top supported models (90% confidence set) for apparent survival (φ) and recapture (*p*) of pied flycatchers.

No.	Model	AICc	ΔAICc	*K*	*w_i_*	Deviance
1	φ_a2(2+: REL)_, *p*_a3*s*c_	1082.7384	0	12	0.46579	1058.3874
2	φ_a2*REL_, *p*_a3*s*c_	1083.0954	0.357	13	0.38964	1056.6855
3	φ_a2(2+: REL)_, *p*_a3*s_	1086.9571	4.2187	9	0.05651	1068.7553
4	φ_a2*REL_, *p*_a3*s_	1087.476	4.7376	10	0.04359	1067.2291
5	φ_(a2*c*REL) + s_, *p*_a3*s_	1089.8896	7.1512	14	0.01304	1061.4161
6	φ_a2+ REL_, *p*_a3*s*c_	1091.0027	8.2643	12	0.00748	1066.6517
7	φ_a2(1 yr: HL_F)_, *p*_a3*s*c_	1092.3524	9.614	12	0.00381	1068.0014
8	φ_a2_, *p*_a3*s*c_	1092.6996	9.9612	11	0.0032	1070.403
9	φ_a2*HL_F_, *p*_a3*s*c_	1092.7067	9.9683	13	0.00319	1066.2968
10	φ_a2(1 yr: HL_M)_, *p*_a3*s*c_	1093.4256	10.6872	12	0.00223	1069.0746
11	φ_(a2*HL_F) + s_, *p*_a3*s*c_	1094.3387	11.6003	14	0.00141	1065.8652
12	φ_a2*s_, *p*_a3*s*c_	1094.5591	11.8207	13	0.00126	1068.1492
13	φ_a2(1 yr: REL)_, *p*_a3*s*c_	1094.6914	11.953	12	0.00118	1070.3404
14	φ_a2(1 yr: HL)_, *p*_a3*s*c_	1094.7269	11.9885	12	0.00116	1070.3759
15	φ_(a2*HL_M) + s_, *p*_a3*s*c_	1095.0261	12.2877	14	0.001	1066.5526

Shown are Akaike information criteria corrected for small sample sizes (AICc), difference in AICc with the top-ranked model (ΔAICc), number of estimable parameters (*K*), normalized Akaike weights (*w_i_*), and deviance. Subscripts denote age (a2 and a3 corresponding to two and three age-classes respectively), sex (s) and cohort (c). Covariates for heterozygosity: fledgling (HL), father (HL_F), mother (HL_M) and genetic relatedness of the parents (REL). ‘1 yr:’ and ‘2+:’ denote an effect only present from fledgling to first-year and from first-year onwards, respectively. Symbol ‘*’ denotes interaction, and symbol ‘+’ additive effects.

The variance explained did not differ between a model including heterozygosity at all single loci and a model including HL for juvenile (F_27,506_ = 1.20, p = 0.22) and adult (F_27,506_ = 0.94, p = 0.54) ages suggesting an absence of single loci effects on survival [62].

## Discussion

Using a wild population of a migratory songbird we explored the relationship between HL and apparent survival at different life stages. Contrary to our expectations, HL was not related to survival either in the juvenile or in subsequent adult life stages. Remarkably, however, the best ranked model in the CMR analyses indicated that the genetic relatedness of the parents (a proxy for inbreeding) negatively influenced offspring survival during adult life, but such effect was not apparent from fledgling to recruitment. Although true survival and permanent emigration are confounded in Cormack-Jolly-Seber models [58], our findings showing an effect during the adult stage suggest an actual relationship between the genetic relatedness of the parents and mortality. First, adults show much higher site fidelity rates and much shorter dispersal distances than juveniles, and our study area was large enough to encompass most between-years movements of breeding individuals (mean breeding dispersal distance = 150 m; [64]–[66]). Second, mean apparent survival probabilities of adult birds (55%, see below), were higher than the highest value reported for other populations of the species (e.g. [67]–[69]).

As expected from theory, positive relationships between heterozygosity and survival, an important component of fitness, have been commonly reported in a variety of taxa (amphibians: [38]; mammals: [19], [29]; birds: [18], [31]). Most of these studies have explored survival in early life stages (i.e. embryo, juvenile or up to recruitment) likely due to the logistical constraints in obtaining longitudinal data across the lifespan of individuals in open populations, and also because the magnitude of HFC is expected to decrease with age [11], [20], [70]. However, measuring HFC at a single life-stage and/or monitoring individuals along only a fraction of their lifespans could cause bias in the reported results. In fact, evidence of HFCs with increasing age has been also reported, with old individuals being more sensitive to environmental variation than young ones [71]. In addition, even opposite effects of heterozygosity have been detected at different life stages with negative and positive effects in early and late life, respectively [12], [36], [72], [73]. Similarly, an effect of the parent’s relatedness on the offspring survival only became apparent in our population after recruitment whereas it was undetectable from fledgling to recruitment (see below). Pied flycatchers are long-distance migrants and, given that HFCs explain on average 1% of fitness variation [6] and fledgling recruitment is low (on average, 22% compared to 55% of adult survival in the study years, as estimated from model 8), stochastic factors operating during migration and/or soon after fledging (e.g. severe adverse conditions and/or high rates of predation) may have overridden any detectable effect of heterozygosity on individual survival up to recruitment. The contrasting effects at different life stages found here add to those in the literature and highlight the need of additional work concerning HSC beyond juvenile phases to clarify its impact in wild populations.

Heterozygosity of the genetic parents (as a specific parental effect; see e.g. [74] or their genetic relatedness have been reported to affect offspring survival. In blue tits, parental HL was positively related to recruitment rate [12] whereas in Seychelles warblers, fledgling survival was influenced by the heterozygosity of both the genetic father ([32]; but see [33]) and mother [33]. Our capture-recapture analyses suggest that pairs formed by genetically similar birds raised individuals with low survival prospects after recruiting as breeders. Further, models including an effect of the father heterozygosity were in general better supported than models without any covariate leading support to the idea that there is a genetic effect of the parents on offspring survival (although the effect of mother heterozygosity was less informative). In this regard, the weak association between the offspring heterozygosity and the relatedness of their parents found here could partly explain the lack of a direct relationship between individual HL and survival in our population. Due to the nature of the markers employed (highly polymorphic microsatellites), the probability of having a high HL (i.e. homozygous individuals) may be low even with genetically similar parents, which may obscure a direct HSC.

None of the loci used appear to be linked to functional genes possibly influencing survival. Further, the lack of identity disequilibrium (estimated as g_2_; see methods) suggests that the typed loci are unrelated to inbreeding at the genome level. However, HFCs can occur (and generally do occur; [62]) in the absence of identity disequilibrium: given that traits are usually influenced by many more loci than those typed, slight inbreeding effects are more easily detected on the phenotype (through HFC) than through correlations in the loci [62]. Thus, although a larger panel of markers should apparently report more precise estimates of inbreeding [17], [75], this argument should not be used to invalidate HFC works [62]. In fact, a recent study of a zebra finch population (with low inbreeding variance) has challenged this view by showing that a panel of 11 microsatellites (the mean microsatellite number used in HFC studies; [6]) located across the genome is as informative as a panel of 1359 SNP markers or a 5^th^ generation pedigree [23]. In the same line, Taylor et al. [76] reported a significant correlation between a pedigree-based inbreeding (*f*) and a panel of 13 microsatellites suggesting that a small set of markers is not necessarily uninformative on individual’s *f* even in wild, outbred populations. In open populations, immigration may generate linkage disequilibrium which, together with random mating, cause identity disequilibrium (i.e. inbreeding; [10]). Unfortunately, we could not measure the degree of the relationship between HL and a pedigree-based inbreeding in this study. The rate of immigration to the population is high and given that reliable inbreeding values must be based on at least two generations (i.e. grandparents-grandsons), we knew the identity of nestlings and grandparents in only 15 broods, with the inbreeding value being zero in all cases. An unsurprising result, since mating between first-order relatives seems to be infrequent in natural populations (around 3%; [77]).

More than a decade ago, Keller and Walker [4] highlighted the need of studies exploring the interaction between genetics, environment and fitness. Today, this type of study is still very scarce. Recent work has highlighted the role of heterozygosity on fitness even in large natural populations with apparent absence of inbreeding [21], which could be determined by environmental conditions [33], [37] or, as in the present work, detected at late stages of life. Hence, we emphasize that studies exploring HFCs at different life stages, in populations with different demographic histories and under variable environmental conditions are required to increase our knowledge on the causes of HFCs.

## Supporting Information

Database S1 (XLSX)
